# Kinetics of Neutrophil Subsets in Acute, Subacute, and Chronic Inflammation

**DOI:** 10.3389/fimmu.2021.674079

**Published:** 2021-06-24

**Authors:** Suzanne H. Bongers, Na Chen, Erinke van Grinsven, Selma van Staveren, Marwan Hassani, Roy Spijkerman, Lilian Hesselink, Adèle T. Lo Tam Loi, Corneli van Aalst, Guus P. Leijte, Matthijs Kox, Peter Pickkers, Falco Hietbrink, Luke P. H. Leenen, Leo Koenderman, Nienke Vrisekoop

**Affiliations:** ^1^ Center for Translational Immunology (CTI), University Medical Center Utrecht, Utrecht, Netherlands; ^2^ Department of Trauma Surgery, University Medical Center Utrecht, Utrecht, Netherlands; ^3^ Department of Respiratory Medicine, University Medical Center Utrecht, Utrecht, Netherlands; ^4^ Department of Intensive Care, Radboud University Medical Center, Nijmegen, Netherlands; ^5^ Radboud Center for Infectious Diseases (RCI), Radboud University Medical Center, Nijmegen, Netherlands

**Keywords:** neutrophils, neutrophil subsets, CD16, CD62L, morphology, acute inflammation, subacute inflammation, chronic inflammation

## Abstract

At homeostasis the vast majority of neutrophils in the circulation expresses CD16 and CD62L within a narrow expression range, but this quickly changes in disease. Little is known regarding the changes in kinetics of neutrophils phenotypes in inflammatory conditions. During acute inflammation more heterogeneity was found, characterized by an increase in CD16^dim^ banded neutrophils. These cells were probably released from the bone marrow (left shift). Acute inflammation induced by human experimental endotoxemia (LPS model) was additionally accompanied by an immediate increase in a CD62L^low^ neutrophil population, which was not as explicit after injury/trauma induced acute inflammation. The situation in sub-acute inflammation was more complex. CD62L^low^ neutrophils appeared in the peripheral blood several days (>3 days) after trauma with a peak after 10 days. A similar situation was found in the blood of COVID-19 patients returning from the ICU. Sorted CD16^low^ and CD62L^low^ subsets from trauma and COVID-19 patients displayed the same nuclear characteristics as found after experimental endotoxemia. In diseases associated with chronic inflammation (stable COPD and treatment naive HIV) no increases in CD16^low^ or CD62L^low^ neutrophils were found in the peripheral blood. All neutrophil subsets were present in the bone marrow during homeostasis. After LPS rechallenge, these subsets failed to appear in the circulation, but continued to be present in the bone marrow, suggesting the absence of recruitment signals. Because the subsets were reported to have different functionalities, these results on the kinetics of neutrophil subsets in a range of inflammatory conditions contribute to our understanding on the role of neutrophils in health and disease.

## Introduction

Neutrophils have long been recognized as essential cells of the innate immune system that eliminate invading pathogens and prevent their systemic spread (1–3). However, the neutrophil compartment is heterogenous and consists of different phenotypes with seemingly different functions (4–8). During acute inflammation, such as experimental endotoxemia, in severe trauma and (bacterial) infection, different subsets of neutrophils are found in the circulation (8–11). These subsets can be visualized by CD16 and CD62L expression in flowcytometry (8, 12, 13). CD16, or FcγRIIIB, is a PI-linked cell surface receptor on neutrophils for the Fc region of IgG (14). CD62L, or L-selectin, is a selectin important in cell adhesion, particularly under flow conditions (15). At homeostasis, circulating neutrophils display a high expression of both CD16 and CD62L. However, during inflammation two additional subsets of CD16^low^ cells and/or CD62L^low^ neutrophils can be found in the circulation (4, 5).

It has been reported that in acute inflammation, CD16^low^ immature banded neutrophils are recruited from the bone marrow to the circulation in a process generally known as ‘left-shift’ (13). These CD16^low^ neutrophils exhibit superior anti-bacterial functions, like phagolysosomal acifdification and bacterial containment, compared to CD16^high^CD62L^high^ mature neutrophils found in the circulation during homeostasis (8, 10). CD62L^low^ neutrophils have also been found in the circulation after inflammatory stimuli and display increased nuclear lobulation. These hypersegmented neutrophils exhibit poor bacterial killing and suppress T cell proliferation (8, 13).

By applying metabolic labeling, Tak et al. have provided evidence that CD16^low^ neutrophils are indeed younger cells than circulating CD16^high^CD62L^high^ mature neutrophils (7). Unexpectedly, CD62L^low^ hypersegmented neutrophils are not found to be older than mature CD16^high^CD62L^high^ neutrophils. Moreover, the CD62L^low^ population also clusters separately in proteomic profile (7). The origin of this neutrophil population is currently unknown.

The cellular and functional heterogeneity of neutrophils are of great interest, as this heterogeneity might be involved in the pathogenesis of inflammatory conditions. So far, the kinetics of neutrophil subsets from the acute to subacute to the chronic phase of inflammation have not been described in great detail. These kinetics are of paramount importance, especially in the light of the recently recognized trained immunity of the innate immune system (16). In the present study, neutrophil subsets were studied under conditions ranging from (hyper)acute to a subacute state of inflammation and also examples of chronic inflammation were discussed. We will show that the neutrophil compartment adjusts its response to inflammatory stimuli regarding the deployment of different neutrophil subsets over time.

## Material and Methods

### Healthy Controls

Blood from 23 healthy controls (HC) was obtained *via* the “mini donor service” at the University Medical Center Utrecht (UMCU, Utrecht, the Netherlands). This service provides blood from healthy volunteers for research purposes. Healthy controls gave consent for blood withdrawal and the protocol is approved by the Medical Ethical Committee of the UMCU under study approval number 18/774. Healthy controls were in good health as determined by self-rapportage. Controls were from both sexes and between 18 and 65 years old, as described before (17). See also **Table 1**. Samples from the trauma and COVID-19 cohorts were compared to these healthy controls.

**Table 1:** Baseline characteristics for each included cohort of subjects and patients. Values are presented as medians (IQR) or as percentages.

		Healthy controls	Experimental endotoxemia model (LPS)	Trauma	COVID-19
*Included*		n=23	n=10	n=15	n=41
*Sex*					
	*Female*	33,3%	0,0%	26,7%	34,1%
	*Male*	66,7%	100,0%	73,3%	65,9%
*Age on admission (years)*		24 (23-27)	23 (19-28)	39 (24–62)	65 (55-72)
*Admission details*					
	*Hospital stay (days)*	n.a.	n.a.	17 (10-26)	19 (14-26,5)
	*ICU stay (days)*	n.a.	n.a.	10 (2-13)	11 (8-16)
*Comorbidities*					
	*History of chronic disease*	0,0%	0,0%	40,0%	61,0%

### Experimental Human Endotoxemia (LPS Model)

Blood- and bone marrow samples were obtained from 10 healthy male volunteers between the age of 18–30 years (see **Table 1**), participating in an experimental human endotoxemia study (ABR NL61136.091.17). Six study participant received a first LPS challenge and five of those participants received a second LPS challenge one week later. Material was gathered before LPS challenge (baseline), four hours after the first LPS challenge and four hours after the second LPS challenge. Study approval was obtained by the ethics review board of the Radboud University Medical Center in Nijmegen, the Netherlands. Written informed consent was obtained from all study participants. Subjects underwent a health screening consisting assessment of medical history, physical examination, electrocardiography and hematological laboratory values. Subjects taking prescription drugs were excluded from the study.

The bone marrow sampling was performed by aspiration from the posterior iliac crest under local anesthesia. A total volume of 40 mL was collected per aspiration into syringes prefilled with sodium heparin. Blood samples were drawn from an arterial catheter (radial artery), using sodium heparin as an anticoagulant. The first blood- and bone marrow sample was obtained prior to the first LPS administration (baseline) and the second blood- and bone marrow sample four hours after both the first and the second LPS challenges. The LPS challenges were performed as published previously by Kiers et al. (18). In short, the subjects were infused with 1.5 L hydration fluid during one hour (2.5% glucose/0.45% saline at a continuous rate). Subsequently, the subjects received a single dose of 2 ng/kg bodyweight LPS (US standard reference Escherichia coli O:113, NIH Pharmaceutical Development Section, Bethesda, MD, USA) and were then infused with hydration fluid at a constant rate of 150 mL/h. During the endotoxemia experiments, heart rate, blood pressure and the course of LPS-induced symptoms such as fever, muscle aches and nausea were constantly monitored.

### Trauma Patients

Blood samples were obtained from 15 multitrauma patients. Trauma patients enrolled in this study, were part of a clinical trial performed at the UMCU. The study was approved by the local ethics committee (ClinicalTrials.gov number NCT03489577, ABR 43279). Written informed consent was obtained from all patients or their legal representatives in accordance with the Helsinki Declaration. Patients suffering from multitrauma who were admitted to the intensive care unit (ICU) of the University Medical Center Utrecht with an expected ICU stay of at least 48 hours were included in this study. Exclusion criteria were: <18 or >80 years old, an altered immunological status and pregnancy. In **Table 1** the baseline characteristics of included patients are displayed. Besides the 15 included patients, two additional patients were eligible for inclusion but no informed consent could be obtained and therefore had to be excluded. When patients met the inclusion criteria, the first blood sample was obtained as soon as possible and at least within 12 hours after hospital admission. Subsequent blood samples were obtained at day 3, 6, 10 and 14 or 15 after trauma. Blood samples were drawn in 4 mL sodium heparin tubes. Some patients have missing data points. Missing data is caused by patients either leaving the hospital within the study period or because of in hospital death within the 15 following days after trauma. Relevant clinical data was extracted from the patient files.

### COVID-19 Patients and Bacterial Infection Patient

The 41 included COVID-19 patients and the bacterial infection patient were part of a prospective cohort study conducted in the University Medical Center Utrecht (UMCU, Utrecht, the Netherlands) during the first wave of the COVID-19 pandemic as described in detail elsewhere by Spijkerman et al. (17). For this study, a waiver for formal ethical approval was provided by the institutional medical ethics committee under protocol number 20-284/C. In short, COVID-19 suspected patients were included upon presentation at the emergency department, where also the first 4 mL blood sample was taken in a sodium heparin tube. All patients were tested for the presence of virus by SARS-CoV-2-specific PCR. Patients were categorized as “COVID-19 positive” if PCR results came back positive. For the specific sub-analysis of pre- and post ICU patients in this study, only male and (non-pregnant) female patients were selected from the database, who had at least one sample taken on the COVID-19 ward <7 days after ICU discharge. Nine of these patients also had data from pre-ICU measurements that were analyzed. Unfortunately, no samples could be obtained during ICU stay. Five patients were excluded from the analysis because they did not have a post-ICU measurement within 7 days after ICU discharge or due to in-hospital death. Relevant clinical data was extracted from the patient files. For baseline characteristics, see **Table 1**.

Among patients included in this cohort who eventually tested negative for COVID-19, some patients tested positive for a bacterial infection based on microbiological cultures. One of these patients was selected as example for acute bacterial infection. This specific patient had a clear onset of symptoms of urinary tract infection, a positive microbial culture and a blood sample taken on the same day. This was an exceptional example of the first phase of onset of a bacterial infection.

### Flow Cytometric Analysis

For the experimental endotoxemia volunteers’ samples, the erythrocytes in blood- and bone marrow samples were lysed using isotonic ice-cold lysis buffer (150 mM NH_4_Cl, 10 mM KHCO_3_ and 0.1 mM Na_2_EDTA dissolved in H_2_O; pH of 7.4). Next, leukocytes were washed and resuspended in FACS staining buffer (4 mg/ml human albumin [Sanquin, Amsterdam, The Netherlands] and 0.32% (w/v) sodium citrate in PBS). Blood- and bone marrow samples were stained with a combination of 10 monoclonal antibodies, fixed in 1% PFA and measured on a BD-LSR Fortessa flow cytometer (Becton Dickenson, Mountain View, CA). The following antibody/fluorochrome conjugations were used: anti-CD35-FITC (clone E11), anti-CD64-APC (clone 10,1), anti-CBRM1/5-Alexa Fluor 700 (clone CBRM1/5), anti-CD11b-APC-Alexa Fluor 750 (clone Bear1), anti-CD305 (LAIR-1)-PE (clone DX26), anti-CD14-eF450 (clone 61D3), anti-CD16-Krome Orange (clone 3G8), anti-CD62L-BV650 (clone DREG 56), anti-CD49d-PECy7 (clone G9F10) and anti-CD66b-PerCPCy5.5 (clone G10F5).

Blood samples of trauma patients, (suspected) COVID-19 patients and healthy controls were measured on an AQUIOS CL^®^ “Load & Go” flow cytometer (Beckman Coulter, Miami, FL, USA). The detailed methods are described elsewhere by Spijkerman et al. (17, 19). For the trauma patients, antibody mixes were used containing anti-CD16-Krome Orange (clone 3G8), anti-CD62L-ECD (clone DREG56), CD10-PC7 (clone ALB1), CD35-FITC (clone J3.D3), anti-CD11c-PeCy5.5 (clone BU15), anti-CD66b-PerCPCy5.5 (clone G10F5) and anti-active CD11b-Alexa700 (clone CBRM1/5). In the COVID-19 cohort and for the healthy controls the following antibody panel was used: CD16-FITC (clone 3G8), CD11b-PE (clone Bear1), CD62L-ECD (clone DREG56), CD10-PC5 (clone ALB1) and CD64-PC7 (clone 22).

Some of the trauma- and COVID-19 samples were also sorted on a FACSAria III flow cytometer (Becton Dickenson, Mountain View, CA, USA) for subsets based on CD16 and CD62L expression, to check for morphological characteristics of the nuclei. Antibody conjugates against CD16-PE-Cy7 (clone 3G8) and CD62L-PErCP-Cy5.5 (clone DREC56) were used.

#### Data analysis

The flow cytometry results were analyzed using FlowJo software (FlowJo LLC, Ashland, OR, USA). All flow panels were compensated using single stains and fluorescence minus one (FMO) experiments were done to help determine setting of the gates. Neutrophils were identified based on their specific forward- and side scatter signals and doublets were excluded from the analysis as much as possible. Eosinophils were excluded either based on the distinctive expression of CD66b or CD16 in panels were these markers were both included, otherwise they were identified in the CD16/CD62L plot as being a distinct population negative for CD16. In addition to the FMO’s, we used the CD62L^high^ lymphocyte population in every sample to determine the placement of the CD62L^low^ gate for neutrophils. The CD16^low^ gate was set based on the intersection between CD62L^low^ cells and CD16^low^ cells. By using this method, a reliable quantification of neutrophil subsets based on CD16 and CD62L could be made, despite the differences between the different flow cytometers and settings. Absolute cell counts for every subset could be calculated based on percentages in the gates and total white blood cell counts.

### Neutrophil Morphology

Cytospin slides of the sorted neutrophil populations were prepared and stained with May-Grünwald-Giemsa staining to determine the nuclear lobularity. The nuclear lobe count was determined by manual counting after visualization on an Axioskop 40 microscope (Zeiss, Jena, Germany) with a 40 objective or a 100x oil immersion objective. Nuclear lobes were considered to be separated if the connection between lobes was less than one third of the width of the adjacent lobes. Progenitors could be identified by nuclear morphology as well as a blue cytoplasm and were counted as having one nuclear lobe (20).

### Statistics

Graphpad Prism version 8.3.0 (GraphPad Software LLC, San Diego, CA, USA) was used to analyze data. Data is presented in graphs as individual data points with mean +/- SD and for the longitudinal trauma data as individual data points with medians. For the experimental endotoxemia data, a one-way ANOVA analysis (paired) with a post-hoc Dunnett’s multiple comparisons test was used to compare blood counts of subsets at different time points (baseline, 1^st^ and 2^nd^ challenge). For the bone marrow samples, a two-way ANOVA analysis (paired) with a post-hoc Sidak’s multiple comparisons test was used to compare the presence of multiple subsets in bone marrow at baseline and after the second LPS challenge. For trauma (HC *vs* day 0, HC *vs* day 10) and COVID-19 (HC *vs* pre-ICU, HC *vs* post-ICU) the same analysis was done as described for the endotoxemia blood samples, although unpaired. Statistical significance was accepted at P^*^ ≤ 0.05, P^**^ ≤ 0.01, P^***^≤ 0.001 or P**** <0.0001.

Materials and methods concerning the data on CD11b in neutrophil subsets in post-ICU COVID-19 patients and trauma patients and the data of COPD and HIV patients, serving as reference for chronic inflammatory conditions, are discussed in the **Supplemental Material**.

## Results

### Acute Inflammation Is Associated With Circulatory CD16^low^ Neutrophils

**Figure 1:**
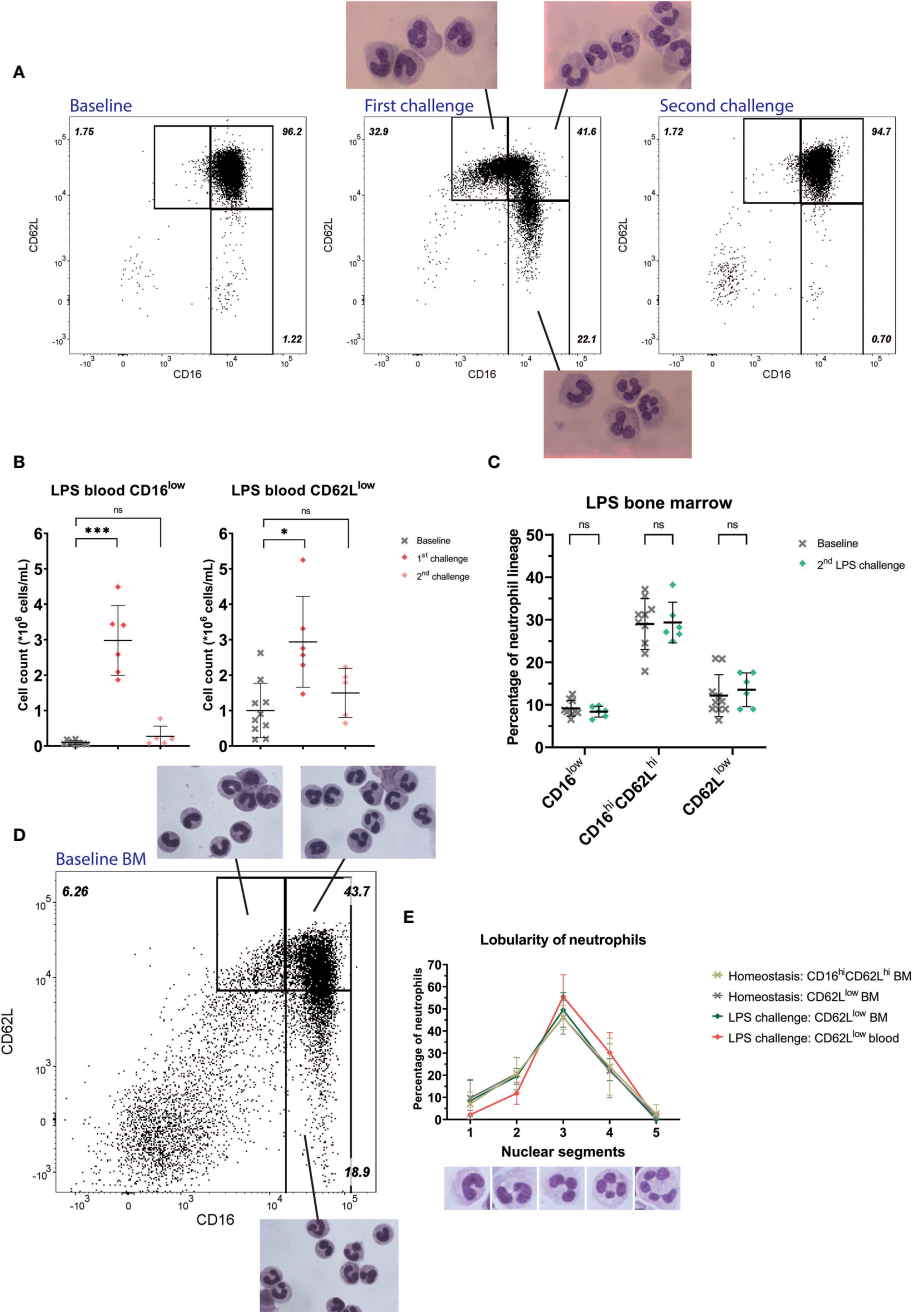
Healthy volunteers undergoing LPS challenge. Representative FACS examples of CD16/CD62L plots of blood from a healthy subject at baseline (first panel), 4 hours after the first LPS challenge with corresponding cell morphology of the specific subsets (second panel) and 4 hours after the second LPS challenge (third panel) **(A)**. Cell counts of CD16^low^ neutrophils (first graph) and CD62L^low^ neutrophils (second graph) at baseline (n=10), after the first (n=6) and second (n=5) LPS challenge. Lines are means with SD. A one-way ANOVA analysis (paired) with a post-hoc Dunnett’s multiple comparisons test was used to test significance **(B)**. Percentages of neutrophil subsets in bone marrow (BM) and after the second LPS challenge. Lines are means with SD. A two-way ANOVA analysis (paired) with a post-hoc Sidak’s multiple comparisons test was used to test significance **(C)**. Representative FACS example of CD16/CD62L plot of bone marrow from a healthy volunteer at baseline with the corresponding cellular morphology for the different subsets **(D)**. Percentages of nuclear lobes of CD62L^low^ neutrophils (ranging from 1 to 5) during homeostasis in bone marrow and during LPS challenge in bone marrow and blood. Lines are means with SD **(E)**. Significance is displayed in graphs as ns, not significant, P^*^ ≤ 0.05 or P^***^≤ 0.001.

Circulatory neutrophils from healthy controls (HC) mainly displayed uniformly high expression of CD16 and CD62L (**Figures 1A** “Baseline” and **1B**). CD16^low^ neutrophils were virtually absent (mean counts: 0.09*10^6^ cells/mL, SD: 0.06*10^6^ cells/mL), whereas CD62L^low^ counts were low but variably present between individuals (mean counts: 1.00 *10^6^ cells/mL, SD: 0.77*10^6^ cells/mL). After LPS administration, CD16^low^ counts (mean: 2.98*10^6^ cells/mL, SD: 0.99*10^6^ cells/mL, P= 0.0007) and CD62L^low^ counts (mean: 2.94*10^6^ cells/mL, SD: 1.28*10^6^ cells/mL, P=0.045) were significantly higher in peripheral blood (**Figures 1A** “First challenge” and **1B**) (21). CD16^low^ neutrophils displayed characteristic immature banded nuclear morphology, whereas nuclei of CD62L^low^ neutrophils were hypersegemented (**Figure 1A** “First challenge”) (21).

**Figure 2:**
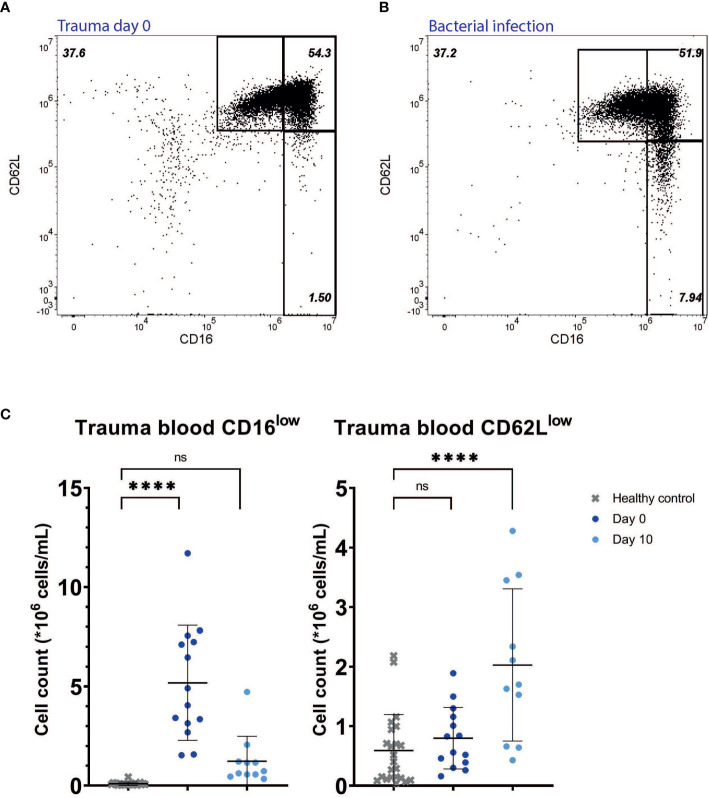
Multitrauma patients. Representative FACS examples of CD16/CD62L plot of blood from a multitrauma patient right after admission (day 0) **(A)**, and from a patient suffering from a bacterial infection at the first day of symptoms **(B)**. Cell counts of CD16^low^ neutrophils (left panel) and CD62L^low^ neutrophils (right panel) in healthy control (HC) blood (n=23) and in blood from trauma patients at day 0 (n=14) and day 10 (n=11) **(C)**. Lines are means with SD. A one-way ANOVA analysis (unpaired) with a post-hoc Dunnett’s multiple comparisons test was used to test significance. Significance is displayed in graphs as ns, not significant or P**** <0.0001.

Trauma patients at the day of hospital admission (day 0) were characterized by the presence of CD16^low^ neutrophils in the bloodstream within hours after injury (mean: 5.18*10^6^ cells/mL, SD: 2.90*10^6^ cells/mL, P<0.0001) when compared to HC (mean: 0.09*10^6^ cells/mL, SD: 0.08*10^6^ cells/mL). CD62L^low^ neutrophil counts were somewhat higher early after injury compared to HC, but this did not reach statistical significance (mean 0,80*10^6^ cells/mL, SD: 0.52*10^6^ cells/mL, P=0.67) (**Figures 2A, C**). Furthermore, in the blood of a patient with a diagnosed bacterial infection early after hospitalization, a similar composition of CD16^low^ and CD62L^low^ neutrophils was recruited to the circulation in this acute phase after onset of infection (**Figure 2B**).

### Subacute Inflammation Is Characterized by the Presence of Increasing Amounts of Activated CD62L^low^ and the Absence of CD16^low^ Neutrophils

**Figure 3:**
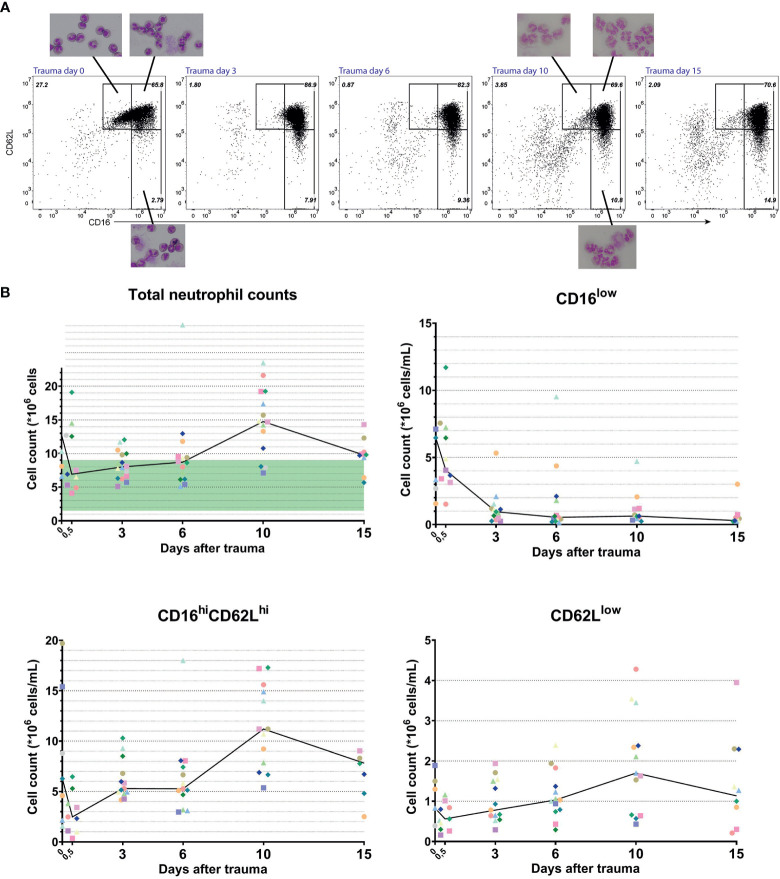
Longitudinal data from multitrauma patients. Representative FACS examples of CD16/CD62L plot of blood from a multitrauma patient. From left to right: at admission (day 0), 3 days, 6 days, 10 days and 15 days after admission to the hospital **(A)**. Kinetics of cell counts over time of all circulating neutrophils (upper left panel), CD16^low^ neutrophils (upper right panel), mature CD16^high^/CD62L^high^ neutrophils (lower left panel) and CD62L^low^ neutrophils (lower right panel). Data from a unique individual is represented by a unique color. A line is plotted through the medians of every time point. In the upper left panel, the normal range of blood counts for neutrophils is marked by a green area ranging from 1,5 to 9*10^6^ cells/mL **(B)**.

The high numbers of CD16^low^ neutrophils found in the circulation early after trauma were not found during the following days (**Figures 2A** and **3A, B** upper right panel). The CD16^low^ counts 10 days after trauma were not significantly increased in the circulation compared to HC (mean: 1.23*10^6^ cells/mL versus 0.09*10^6^ cells/mL for HC, P=0.13). CD62L^low^ neutrophil counts, on the other hand, were significantly elevated after 10 days (mean: 2.03*10^6^ cells/mL versus 0.59*10^6^ cells/mL for HC, P<0.0001) (**Figure 2B**).

Next, we followed the kinetics of the different neutrophil subsets in developing inflammation. The same multitrauma patients were followed in time and their neutrophil subsets were determined at day 0 (right after admission) or day 0,5 (several hours after admission), 3, 6, 10 and day 14 or 15 after admission. As can be seen in **Figure 3B** (upper left panel), total neutrophil counts were above the normal range early after trauma (day 0) and again late after trauma (at days 10–15) (22). Neutrophilia at day 0 is mainly caused by high counts of CD16^low^ neutrophils and decreased quickly within hours, as can be seen in the same graph (day 0 *vs* day 0,5 and day 3). Interestingly, within 3 days after multitrauma, the CD16^low^ population virtually disappeared from the circulation. The CD62L^low^ population on the other hand, started increasing from day 3 onward and reached its peak at day 10 (**Figures 3A, B**, upper- and lower right panels). This increase in CD62L^low^ neutrophils in combination with a surge in CD16^high^CD62L^high^ neutrophils were responsible for the second phase of neutrophilia.

CD16^low^ neutrophils showed a clear band-shaped nucleus. Neutrophils found at later time points (i.e. day 10) were harder to examine on cytospin slides, because they were more fragile (**Figure 3A**). Nevertheless, CD62L^low^ cells that could be observed indeed expressed more lobes than mature CD16^high^CD62L^high^ cells. In addition, the presence of vacuoles in the nucleus and cytoplasm was noteworthy. This was also seen in CD62L^low^ neutrophils at other time points after trauma (data not shown).

**Figure 4:**
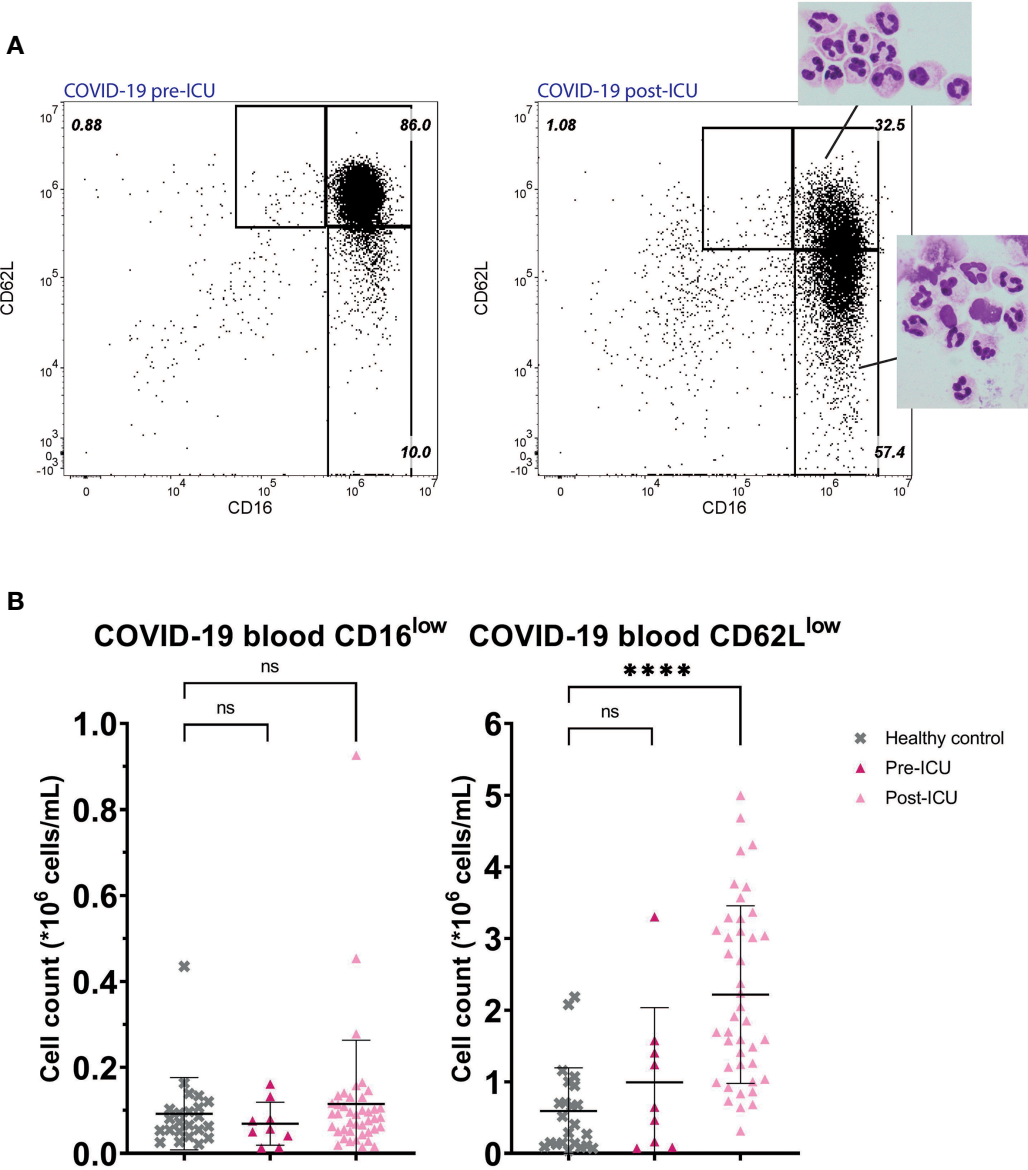
COVID-19 patients pre- and post-ICU. Representative FACS examples of CD16/CD62L plot of blood from a hospitalized COVID-19 patient before admission to the ICU (left panel) and from the same patient post-ICU with corresponding nuclear morphology of the subsets (right panel) **(A)**. Cell counts of CD16^low^ neutrophils (left panel) and CD62L^low^ neutrophils (right panel) in healthy control (HC) blood (n=23) and in blood from COVID-19 patients pre-ICU (n=9) and post-ICU (n=41) **(B)**. Lines are means with SD. A one-way ANOVA analysis (unpaired) with a post-hoc Dunnett’s multiple comparisons test was used to test significance. Significance is displayed in graphs as ns, not significant, P**** <0,0001.

A similar situation to trauma at day 10 was found in post-ICU patients suffering from COVID-19 (**Figure 4**). These patients who returned from the ICU also exhibited a pronounced increase in circulating CD62L^low^ neutrophils (mean: 2.22*10^6^ cells/mL, SD: 1.24*10^6^ cells/mL, P<0.0001). The CD62L^low^ cells found in these post-ICU patients showed hypersegmentation and fragility (**Figure 4A**, second panel). CD62L^low^ cells were not significantly present before ICU admission (mean: 0.99*10^6^ cells/mL, SD: 1,04*10^6^ cells/mL, P=0.54). In COVID-19 patients CD16^low^ neutrophils were not increased pre-ICU (mean: 0.07*10^6^ cells/mL, SD: 0.05*10^6^ cells/mL, P=0.85) nor were they post-ICU (mean: 0.12*10^6^ cells/mL, SD: 0.15*10^6^ cells/mL, P=0.71) compared to healthy controls.

We also checked for the activation status of CD62L^low^ neutrophils by measuring de median fluorescent intensity (MFI) of CD11b in the different subsets of trauma patients at day 0 and 10 and post-ICU COVID-19 patients. Compared to the mature CD16^high^CD62L^high^ subset (mean MFI: 315444, SD: 140583), CD62L^low^ neutrophils were higher in CD11b in trauma patients at day 0 (mean MFI: 611250, SD: 219799, P= 0,027). This was also the case in trauma patients at day 10 when comparing mature CD16^high^CD62L^high^ neutrophils (mean MFI: 254296, SD: 161328) to CD62L^low^ cells (mean MFI: 286422, SD: 147249, P=0.037). Results are shown in **Supplemental Figure 2A**. In post-ICU COVID-19 patients, the increase in CD11b expression of CD62L^low^ neutrophils (mean MFI: 2262721, SD: 699731) was also significant compared to CD16^high^CD62L^high^ neutrophils (mean MFI: 1720767, SD: 681105, P <0,0001, **Supplemental Figure 2B**).

### CD62L^low^ Neutrophils Are Found in Bone Marrow During Homeostasis, but With Less Nuclear Segmentation

In healthy bone marrow, neutrophil progenitors were present, consisting of promyelocytes, myelocytes and metamyelocytes (data not shown) (23) and also mature CD16^high^CD62L^high^ neutrophils were present. In addition, both CD16^low^ neutrophils and CD62L^low^ neutrophils could be found in bone marrow during homeostasis (**Figure 1C** “Baseline”). We scored the lobularity of CD62L^low^ neutrophils in the bone marrow and compared this to CD62L^low^ neutrophils from the circulation after LPS administration. In contrast to the increased lobularity found in CD62L^low^ cells from the circulation after LPS administration, trauma and COVID-19 infection, the lobularity of CD62L^low^ neutrophils in the bone marrow overlapped with those of normal mature CD16^high^CD62^high^ neutrophils in bone marrow (**Figures 1D, E**).

### Lack of Recruitment of CD16^low^ and CD62L^low^ Neutrophils During a Second LPS Challenge Despite the Presence of CD16^low^ Neutrophils in the Bone Marrow

As a model system for repeated inflammatory insults, we monitored neutrophil subsets after a second LPS challenge seven days after the first. Four hours after the second LPS challenge the neutrophil compartment was remarkably similar to baseline samples. Neither CD16^low^ (mean: 0.27*10^6^ cells/mL, SD: 0.29*10^6^ cells/mL, P=0.208) nor CD62L^low^ neutrophil subsets (mean: 1.50*10^6^ cells/mL, SD: 0.69*10^6^ cells/mL, P=0.308) were significantly recruited to the circulation when compared to baseline (**Figure 1A** “Second challenge” and **1B**) (24). We also monitored the bone marrow four hours after the second LPS challenge and found CD16^low^ neutrophils were present in the bone marrow in the same proportions as during homeostasis (**Figure 1C**). These data suggest CD16^low^ neutrophils fail to be recruited from the bone marrow to the circulation during this second inflammatory stimulus.

### COPD and HIV Patients as Models for Chronic Inflammation Are Characterized by the Absence of CD16^low^ and CD62L^low^ Neutrophils in the Circulation

In order to study the presence of neutrophil subsets in a low-grade chronic inflammatory condition, we chose to characterize COPD patients suffering from neutrophilic inflammation (25, 26) and therapy naïve HIV infected patients. Similarly as found during the later time-points after multitrauma, hardly any immature CD16^low^ neutrophils were found in the circulation of COPD (**Supplementary Figure 1A**) and HIV patients (**Supplementary Figure 1B**). CD62L^low^ neutrophils were similarly low in the circulation of these patients. Overall, the subset profiles of these COPD and HIV patients seemed comparable to those of healthy subjects. The severity of the COPD patients ranged between GOLD I-IV, but no significant differences were found between patients in different GOLD stages (data not shown).

## Conclusion and Discussion

Neutrophil CD16/CD62L-based subsets were studied and compared under different inflammatory conditions, ranging from (hyper) acute to subacute and chronic inflammation. The distribution patterns and kinetics of CD16^low^, CD16^high^CD62L^high^ and CD62L^low^ neutrophils in the different types of inflammation indicated a very dynamic control of these cells in the peripheral blood. In addition to conventional mature CD16^high^CD62L^high^ neutrophils, CD16^low^ neutrophils with a band shaped nucleus were predominantly recruited to the circulation during the hyper acute phase after an inflammatory insult. The occurrence of young band-form neutrophils is a well-known phenomenon and generally referred to as a “left shift” (27). These CD16^low^ banded neutrophils were only present for a couple of hours to maximally a few days during acute inflammation after trauma and LPS challenge and were conspicuously absent from the circulation thereafter, as shown in subacute trauma and COVID-19 patients as well as in chronic conditions such as COPD and HIV infection (treatment naïve). Thus, the CD16^low^ cells seem to be present only in the very acute moment of an inflammatory insult and disappear within approximately a day (as can be seen in the longitudinal trauma data). Since COVID-19 patients were already infected for at least a few days before their blood was analyzed, our data set cannot ascertain whether CD16^low^ cells were present or absent in the acute phase of the disease. The CD62L^low^ subset showed more complex kinetics. These cells appear in the circulation during acute inflammation evoked by experimental endotoxemia, but to a much lesser extent at the day of admission after trauma. During the subacute follow-up after trauma these cells increased in circulating counts and showed the typical hypersegmented nuclei. When inflammation is chronic, like is the case in COPD and HIV infection, the neutrophil subset profile returned to normal, indicating an attempt to restore a balance in the neutrophil compartment. The seemingly normalized situation in chronic disease could be based on the same mechanism of action driving the desensitization to a second LPS challenge, as found in the human experimental endotoxemia model. However, the observation that the subset profile in the blood returned to normal, does not exclude the possibility that neutrophil numbers could remain elevated in the tissues during chronic inflammation.

Circulatory CD62L^low^ neutrophils were higher in activation markers such as CD11b, as has been demonstrated before (28). In this study no functional capacities of the neutrophil subsets were tested. However, Hesselink et al. previously published data on the phagocytosis and phagolysosomal acidification capacity of CD16/CD62L neutrophil subsets in trauma patients (10). These capacities are of major importance in the defense against pathogens. It was shown that the CD16^low^ subset displayed a better phagolysosmal acidification capacity when compared to “normal” CD16^high^/CD62L^high^ neutrophils. The CD62L^low^ subset on the other hand showed a trend towards a reduced acidification capacity (10). The actual bacterial containment of the different subsets can be tested in a containment assay with *S. aureus*, like the one described by Van Grinsven et al. (29). In this assay CD62L^low^ cells of volunteers after LPS administration were previously shown to exhibit decreased antibacterial function (8, 10). Even more interesting, CD62L^low^ neutrophils were shown to have more immunoregulatory characteristics that could be important for keeping balance in the immune response (7, 13) and maybe even recovery of tissues (30). The presence of high counts of CD62L^low^ neutrophils with immunosuppressive properties in the circulation might also have a flipside. It is known that trauma patients are highly susceptible to infections (31). The presence of high counts of CD62L^low^ neutrophils during the days to weeks following severe trauma might play a significant role in the infectious prone state these patients are in (9). The first infectious complications arise at the end of the first week after trauma (32). This coincides with the rising counts of CD62L^low^ cells during the first week after trauma, peaking at day 10. However, this observation needs to be further explored to investigate whether there is a (causal) relationship between the occurrence of CD62L^low^ cells and the presence of infections. In addition, it is also possible that trauma patients are desensitized for additional stimuli after the first big inflammatory hit of the initial trauma and are therefore not able to mount an adequate response to invading pathogens. These patients often undergo surgeries in the days after initial trauma, causing extra stress on the innate immune system. In the endotoxemia model we showed that after a second challenge with LPS, a week after the first challenge, less CD16^low^ cells were recruited to the circulation, like was shown before. Also the CD62L^low^ neutrophil counts were lower in blood during a second challenge, but this difference was not as pronounced as seen for the CD16^low^ cells when compared to the first challenge (24). It appears the same inflammatory insult evokes a less pronounced response after a primary challenge. This situation is comparable to the situation of trauma patients, who also undergo multiple inflammatory hits. Two explanations for the lack of CD16^low^ neutrophil recruitment to the circulation could be: 1) the bone marrow was devoid of CD16^low^ cells after the first LPS challenge and not yet replenished, or 2) CD16^low^ neutrophils were present in the bone marrow but failed to be recruited. We showed that the same percentage of CD16^low^ cells was still present in the bone marrow after the second LPS stimulus, compared to baseline bone marrow samples. In addition, it is demonstrated that there is notably less change in plasma cytokine and chemokine levels after re-challenge (25, 33). This suggests an adaptation of the innate immune response after multiple inflammatory stimuli. Although historically a memory immune response was reserved for the adaptive immune system, it has recently become clear that even the innate immune system can adapt its response. This has been coined ‘trained immunity’ by Netea et al. in case of an increased responsiveness to a secondary challenge (26) and ‘tolerance’ or ‘immunoparalysis’ in case of an attenuated responsiveness to a secondary challenge (33, 34). Immunoparalysis might account for the susceptibility of trauma patients to secondary infections (34–36).

The origin of CD62L^low^ cells appearing in the circulation during inflammation is still unknown (37). We demonstrated that CD62L^low^ neutrophils were present in de bone marrow during homeostasis and also in small amounts in the circulation of healthy subjects. The CD62L^low^ neutrophils in the bone marrow did not show pronounced hypersegmentation of the nucleus, in contrast to the circulating CD62L^low^ neutrophils found after experimental endotoxemia, subacute trauma and in post-ICU COVID-19 patients. Because the lobularity of CD62L^low^ neutrophils in the bone marrow is less, it is unlikely that all these cells are old circulatory CD62L^low^ neutrophils returning to the bone marrow to undergo apoptosis (38). Whether the CD62L^low^ neutrophils in the bone marrow are the precursors of the CD62L^low^ neutrophils in the circulation or whether these are different subsets, remains to be investigated. We have previously demonstrated that the circulatory CD62L^low^ neutrophils cluster separately from CD16^low^ and CD16^high^CD62L^high^ neutrophils after analysis of proteomic profiling data, suggesting a separate CD62L^low^ neutrophil lineage (7). If the CD62L^low^ neutrophils from the bone marrow are their precursors, this would imply that the increased lobularity and expression of activation markers in the circulation needs to be achieved shortly after the inflammatory stimulus, since we found these cells in the circulation within three hours after LPS (13). Alternatively, CD62L^low^ neutrophils in the circulation are separate from their bone marrow counterparts and might be cells which shed CD62L during extravasation (20), got activated in the (inflamed) tissue and returned from the tissues to the circulation (39). The increased levels of activation markers of CD62L^low^ cells in the circulation during inflammatory conditions, as shown in this work and previously (28), is in line with this hypothesis.

This study also has its limitations. Different flow cytometers were used to gather the data for this study. This comes with a few challenges regarding differences in settings, different antibody panels and different machine properties. However, we found a method to reliably gate the different CD16/CD62L neutrophil subsets based on CD62L^high^ lymphocyte populations. This made it possible to still make a valid comparison between different datasets. Another challenge was the samples size in some of the used datasets. Especially the LPS cohort had a relatively small sample size of n=10. This might have caused a lack of statistical power. However, the human experimental endotoxemia model is performed in a highly controlled setting and therefore is not influenced by possible confounders like age, comorbidities or effects of treatment, like is the case for trauma and COVID-19 patients. The timing of sample collection is also a point of discussion, especially in the acute moment after trauma. Since the CD16^low^ subset is only present in the very acute moment and the cell counts of this subset already start to decline within the same day after sever injury, it is vital that the sample is taken as soon as possible. However, in our data there is some variation in timing of the first sample. Some samples are taken in the trauma bay right after arrival in the hospital (day 0), but for some patients direct blood sampling was not possible and a blood sample was taken a few hours after admission. These samples are pooled in the group “day 0.5”. However, this might have caused some variation in the data, since not all samples are taken at the same time point after trauma. During the follow up of the trauma patients, different confounders were introduced due to infectious complications, interventions or drug therapy (corticosteroids, antibiotics e.a) that could have influenced our data. It is known that surgical interventions and mechanical ventilation have pronounced effects on the innate immune system (40, 41). Severely injured patients often undergo a combination of these procedures, probably causing at least part of the variation in the longitudinal data. Nevertheless, the temporal pattern of the neutrophil subset response to trauma is still evident.

In summary, immature banded CD16^low^ neutrophils only appear in the circulation during the hyper acute phase of acute inflammation, whereas activated hypersegmented CD62L^low^ neutrophils first appear after the acute phase and rise during the consecutive days and continue to be present over a prolonged period in subacute inflammatory conditions. During chronic inflammatory diseases, the subset profile normalizes again, suggesting a restoration of balance or setting of ‘tolerance’. Our results on the appearance and kinetics of neutrophil subsets in a range of inflammatory conditions contributes to the understanding of (im)balances in the innate immune response.

## Data Availability Statement

The datasets presented in this article are not readily available, however the data can be shared by the corresponding author upon reasonable request. Requests to access the datasets should be directed to NV, N.Vrisekoop@umcutrecht.nl.

## Ethics Statement

The studies involving human participants were reviewed and approved by Medical Research Ethics Committee (MREC) Utrecht and CMO region Arnhem-Nijmegen. The patients/participants provided their written informed consent to participate in this study.

## Author Contributions

AL, GL, MK, PP, FH, LH, LL, LK, and NV contributed to conception and design of the study. CA, SB, EG, SS, MH, RS, LH, AL, and GL performed and analyzed experiments. SB, NC, and NV wrote the first draft of the manuscript. All authors contributed to the article and approved the submitted version.

## Funding

This work was supported by a personal fellowship from the China Scholarship Council (CSC: 201406170046) to NC. Part of this research was carried out in the framework of the Top Institute Pharma project T1-108 ‘Acute and chronic inflammatory responses-COPD and smoking’, with partners University Medical Center Groningen (UMCG), University of Groningen (RUG), GRIAC Research Institute Groningen, University Medical Center Utrecht (UMCU), Nycomed BV, GlaxoSmithKline and Foundation TI Pharma. The funder was not involved in the study design, collection, analysis, interpretation of data, the writing of this article or the decision to submit it for publication.

## Conflict of Interest

The authors declare that the research was conducted in the absence of any commercial or financial relationships that could be construed as a potential conflict of interest.
