# Supplementary material for: Kinetics of Neutrophil Subsets in Acute, Subacute, and Chronic Inflammation
Source: Front Immunol. 2021 Jun 24;12:674079. doi: 10.3389/fimmu.2021.674079 (PMC8265311; doi:10.3389/fimmu.2021.674079)
Supplement: Supplementary file 1 [file DataSheet_1.pdf]

Supplementary Material

Supplement 1: Examples of chronic inflammatory disease, COPD and HIV infection.

COPD Patients

Data from COPD patients was collected as part of a cross-sectional study performed by the University Medical Center Utrecht (UMCU, Utrecht, the Netherlands) and University Medical Center Groningen (UMCG, Groningen, the Netherlands). Trial register numbers are NCT00850863 and NCT00807469 (www.clinicaltrials.gov). The study design has been described elsewhere (1). A total of 50 patients were included. Patients had a smoking history of more than ten pack years and a ratio of FEV1 to forced vital capacity of maximum 70% after bronchodilator use. COPD patients ranging from GOLD stage 1 to 4 were included. GOLD stages were based on the criteria of the global initiative for chronic obstructive lung disease from 2013 (https://goldcopd.org). Patients with a history of other inflammatory diseases, including asthma and active infections, were excluded as were patients treated with antibiotics and/or corticosteroids up to 8 weeks before inclusion.

COPD patient samples were stained with an anti-CD16 (clone 3G8) and anti-CD62L (clone DREG56) antibody mix and measured on a FACSCalibur (Becton Dickenson, Mountain View, CA, USA).

When compared to age- and smoking status-matched healthy controls as described in (1), no significant difference was found in percentages of $CD16^{low}$ (mean: 1,70%, SD: 2,62%, P=0,161) and $CD62L^{low}$ neutrophil subsets (mean: 13,91%, SD: 14,34%, P=0,712). Therefore, these data are not shown, but instead a representative example of the subset distribution is displayed in Supplemental Figure 1A which is comparable to the subset distribution of healthy subjects.

HIV patients

The dataset of HIV patients consists of four patient samples. Patients were not treated with anti-retroviral therapy. Peripheral blood samples were obtained from individuals in the context of standard diagnostic care. Residual blood not used for the standard diagnostic tests was used for research, with Informed Consent from the patient according to protocols of the UMC Utrecht.

HIV patient samples were stained with 10 fluorescently labeled antibodies against CD123 (FITC, clone 6H6), CD14 (APC, RMO52), CD16 (Krome Orange, clone 3G8), CD3 (PB, clone UCHT1), CD4 (AF750 clone 13B8.2), CD8 (APC-AF700 clone B9.11), CD56 (PE-Cy5.5 clone N901), CD20 (PE-Cy7 clone B9E9), CD193 (PE clone 5E8) and CD62L (BV650 clone DREG56).

Because of the very small patient population, this dataset was only used for orientation purposes and no statistics were performed. However, the subset profiles of these patients did resemble those of healthy controls, as can be seen from the example in Supplemental Figure 1B.

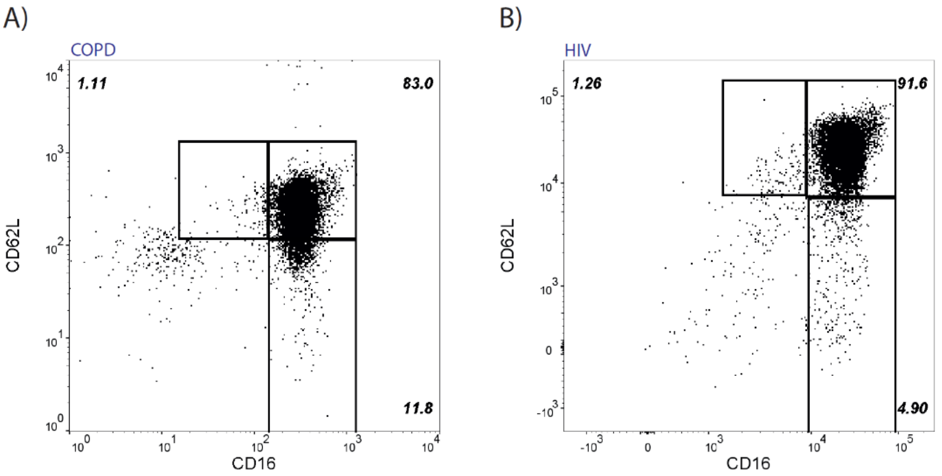

Supplementary Figure 1: Representative examples of patients with a chronic inflammatory disease (COPD and HIV). Representative FACS example of CD16/CD62L plot of blood from a COPD patient in a stable situation (no exacerbation) (**A**) and an example from a treatment naïve HIV patient (**B**).

References

1. Lo Tam Loi AT, Hoonhorst SJM, Franciosi L, Bischoff R, Hoffmann RF, Heijink I, Van Oosterhout AJM, Boezen HM, Timens W, Postma DS, et al. Acute and chronic inflammatory responses induced by smoking in individuals susceptible and non-susceptible to development of COPD: From specific disease phenotyping towards novel therapy. Protocol of a cross-sectional study. *BMJ Open* (2013) **3**:1–10. doi:10.1136/bmjopen-2012-002178

Supplement 2: Expression of activation marker CD11b in $CD16^{high}CD62L^{high}$ and $CD62L^{low}$ subsets in trauma and COVID-19 patients.

Paired T-tests were used to compare median fluorescence intensity (MFI) of activation marker CD11b in the $CD62L^{low}$ subsets to the expression in the $CD16^{high}CD62L^{high}$ subset of post-ICU COVID-19 patients and in trauma patients at day 0 and day 10. Statistical significance was accepted at P* ≤ 0.05, P** ≤ 0.01, P***≤ 0.001 or P**** <0.0001.

The results of these analyses are shown in Supplemental Figure 2 and discussed in the main text of the article.

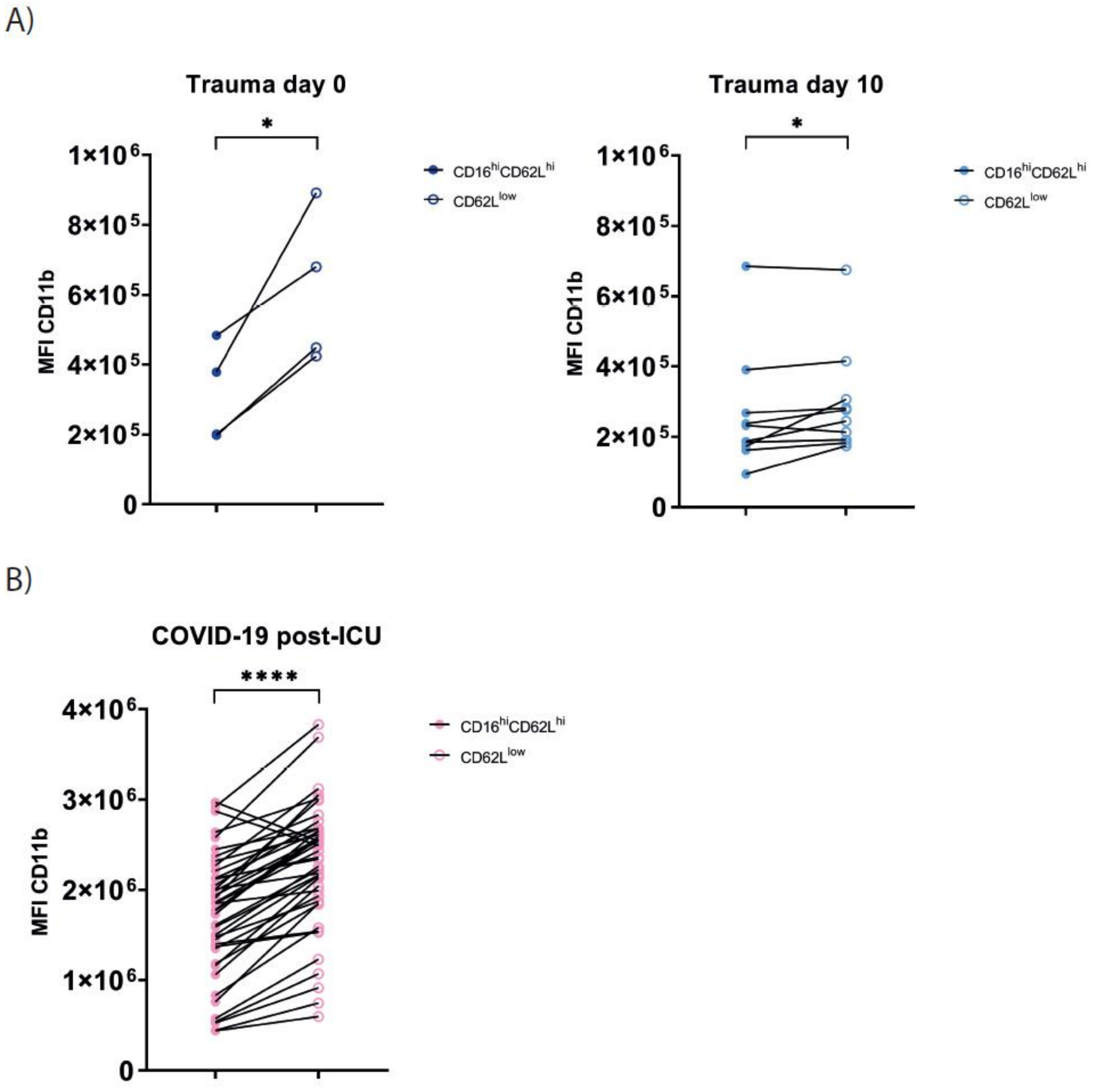

Supplementary Figure 2: Expression of activation marker CD11b on $CD16^{high}CD62L^{high}$ and $CD62L^{low}$ subsets in trauma patients at day 0 and day 10 (**A**) and in post-ICU COVID-19 patients (**B**). Paired T-tests were performed to test significance. Significance is displayed in graphs as $P^{*} \leq 0.05$ or $P^{****} \leq 0.0001$.
